# Flow virometry for process monitoring of live virus vaccines-lessons learned from ERVEBO

**DOI:** 10.1038/s41598-021-86688-z

**Published:** 2021-04-01

**Authors:** Geoffri Ricci, Kevin Minsker, Austin Kapish, James Osborn, Sha Ha, Joseph Davide, Joseph P. Califano, Darrell Sehlin, Richard R. Rustandi, Lawrence W. Dick, Josef Vlasak, Timothy D. Culp, Andreas Baudy, Edward Bell, Malini Mukherjee

**Affiliations:** 1 Vaccines Process Development and Commercialization, Merck & Co., Inc., 770 Sumneytown Pike, WP 42-3, West Point, PA 19486 USA; 2 Vaccines Analytical Research and Development, Merck & Co., Inc., West Point, PA USA; 3 Vaccines Process Development, Merck & Co., Inc., West Point, PA USA; 4 Safety Assessment and Laboratory Animal Resources, Merck & Co., Inc., West Point, PA USA

**Keywords:** Biological techniques, Flow cytometry, High-throughput screening

## Abstract

Direct at line monitoring of live virus particles in commercial manufacturing of vaccines is challenging due to their small size. Detection of malformed or damaged virions with reduced potency is rate-limited by release potency assays with long turnaround times. Thus, preempting batch failures caused by out of specification potency results is almost impossible. Much needed are in-process tools that can monitor and detect compromised viral particles in live-virus vaccines (LVVs) manufacturing based on changes in their biophysical properties to provide timely measures to rectify process stresses leading to such damage. Using ERVEBO, MSD’s Ebola virus vaccine as an example, here we describe a flow virometry assay that can quickly detect damaged virus particles and provide mechanistic insight into process parameters contributing to the damage. Furthermore, we describe a 24-h high throughput infectivity assay that can be used to correlate damaged particles directly to loss in viral infectivity (potency) in-process. Collectively, we provide a set of innovative tools to enable rapid process development, process monitoring, and control strategy implementation in large scale LVV manufacturing.

## Introduction

Traditional LVV manufacturing uses mammalian cellular substrates for virus production^1–5^. The resulting virus harvest then undergoes a series of purification and concentration steps to generate the bulk drug substance which is typically frozen. Weeks or months later the bulk drug substance will be thawed, formulated and filled into vials which may then be lyophilized to generate the stable drug product (DP) for patient use. For a typical large-scale LVV manufacturing process, the time from initial virus infection of cells to vial filling is several months. Across all of these processing steps, it is important to preserve the critical quality attributes (CQA) of the virus particles, which, for most LVVs, are infectivity or antigenicity, or both. Potency is determined by infectivity of the virus particles in a known host cell and is often measured using a plaque forming assay by counting the number of plaque forming units (pfu) or by a TCID50 assay. Antigenicity, if required, is usually measured using an ELISA. Both plaque or ELISA are used for release testing of LVV batches at the end of manufacturing a vaccine batch for drug substance (DS) or DP^6–9^. Both of these release assays are time and resource-intensive, resulting in extended lead times of about two weeks or more to obtain results. However, these assays are not designed to detect and respond to any at line variabilities that can affect the individual virus particles being manufactured. Control strategies currently in place for most commercial LVV manufacturing include in line probes to obtain measurements of cellular metabolites like glucose and lactate and monitoring pH and gas profiles of oxygen and CO2. While these measurements provide an indication of changes in the infected cell culture and thereby offer an indirect way to monitor virus particles, they do not provide sufficient direct control or monitoring of virus quality or quantity. Development of process analytical tools (PAT) that can offer innovative ways to directly monitor virus particles at-line are much needed. The benefits such a PAT could offer include developing a strong correlation between total virus particles manufactured in-process and their predicted infectivity and antigen levels in DS. Release assays could then only be used for a final commercial batch release and not for intermediate testing of DS. A second benefit is detection and response to any in-process virus alterations in real time. Conceivably, a suitable PAT intervention for direct virus monitoring can offset some unique vulnerabilities of existing LVV manufacturing, like inadvertent generation of damaged virus particles resulting in out of specification (OOS) DS and costly batch failures.

In addition to the final manufacturing process, scale-down process models are used to establish process parameter ranges for the commercial-scale manufacturing process during process characterization and development [McKnight, N., “Scale-down Model Qualification and Use in Process Characterization.” CMC Strategy Forum. Jan 28, 2013, ICH Q11, “Development and Manufacture of Drug Substances (Chemical Entities and Biotechnological/Biological Entities), May 1, 2012, CBER/CDER/CVM, “Guidance for Industry: Process Validation—General Principles and Practices.” US Food and Drug Administration, Jan 2011]. Experiments using scale-down models investigate the relationship between variability in-process parameters and quality attributes. Key process parameters (KPPs) evaluated during these studies include cell seeding density and virus multiplicity of infection (MOI) as well as temperature and media conditions for the process. These studies, known as “range-finding”, require rapid response to multiple conditions during process characterization and produce far more samples than that is typical of a final manufacturing process. A PAT for high-throughput and immediate, at-line results for viral particle quality and quantity are valuable for rapid response and further iterations of these studies.

However, direct monitoring of virus particles has remained challenging due to their sub-micron sizes. Dynamic light scattering (DLS) as well as flow cytometry have been explored as PAT for direct at-line monitoring of virus particles. Modifications have been made to conventional flow cytometers to enable direct measurement of submicron particles. A number of laboratories have recently reported success in utilizing such customized “flow virometers” as well as newer standard commercial flow cytometers with high-powered violet lasers to detect biological particles smaller than 300 nm, including extracellular vesicles, exosomes, and larger viruses^10–22^. Violet lasers are often utilized as they have the shortest wavelength and thus are scattered with more frequency by sample streams with particles less than or equal to the wavelength. The more scatter produced by running the sample, the easier it is to differentiate from incidental background noise, producing a clean signal. While these standard, commercially available units are advertised as flow cytometers, they can effectively enumerate smaller particles such as viruses and are referred to as flow virometers in this study.

**Figure 1:**
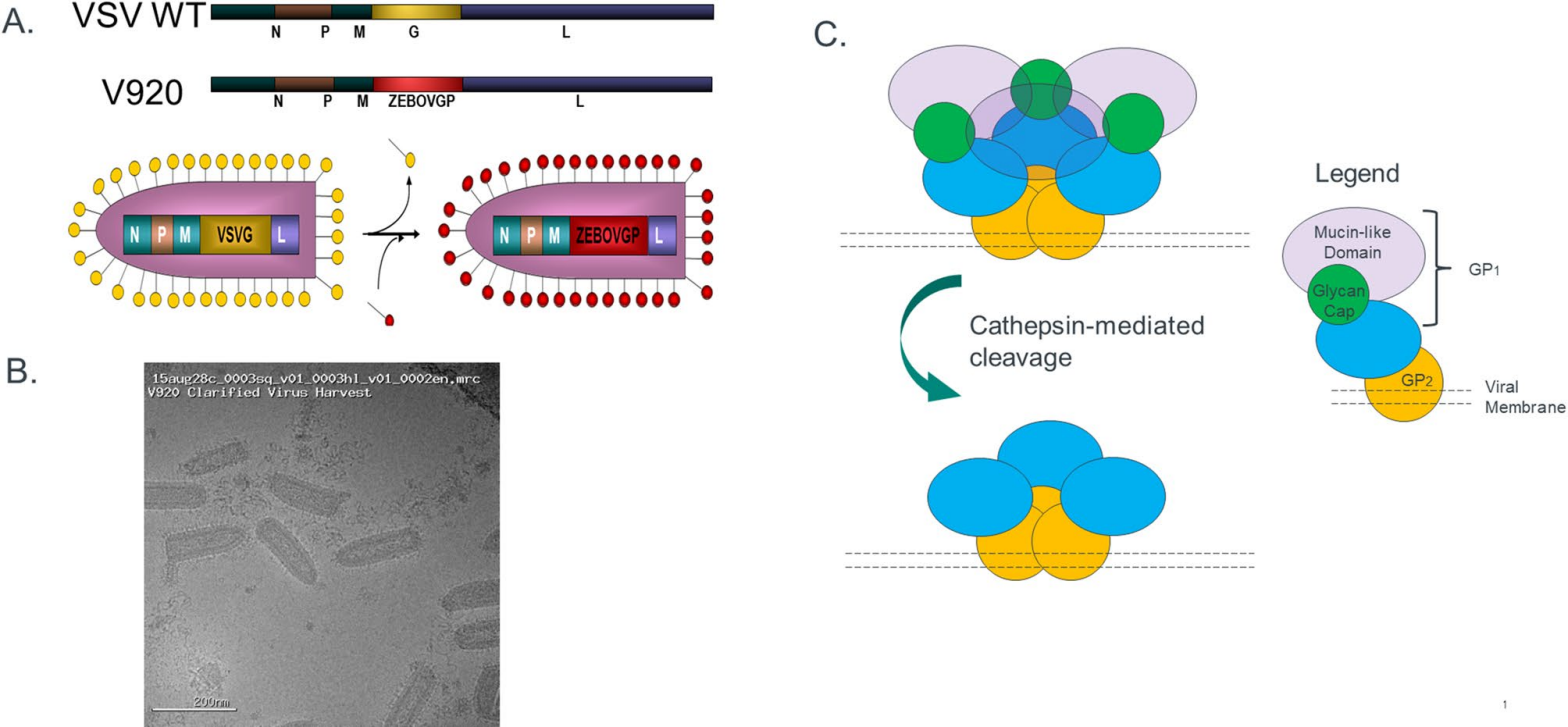
Description of structure and morphology of rVSV-ZEBOV-GP. (**A**) MSD’s Ebola Vaccine was generated by recombinant engineering and genetic swapping of Vesicular Stomatitis Virus (VSV) surface glycoprotein with Ebola virus surface GP1 glycoprotein. (**B**) Cryo-EM of clarified virus harvest material from laboratory samples of rVSV-ZEBOV, scale shown for size reference. (**C**) Model representation of cathepsin-mediated cleavage in natural Ebola surface GP proteins, legend included. The Trypsin treatment performed during processing of ERBEVO is thought to simulate natural viral protein processing, increasing infectivity of the produced virions.

In this study, we first applied flow virometry to characterize ERVEBO (Ebola Zaire Vaccine, Live), the recently approved rVSV-ZEBOV-GP LVV. We then evaluated the effects of relevant process parameters on this product using lab scale studies and the utility of flow virometry in detecting damaged virions early in a scale down model of the manufacturing process. ERVEBO is a live, vesicular stomatitis virus (VSV), genetically engineered to express glycoprotein from Zaire Ebolavirus, to elicit a protective immune response in vaccinated individuals (Fig. 1A, adapted from^23,24^. The VSV virions are roughly bullet-shaped and 70 nm by 200 nm in size based on cryo-EM images (Fig. 1B). The Ebola virus uses multiple post-assembly processing steps to mature the GP1/GP2 complexes^25–27^. These include endosomal proteolysis as well as cathepsin-mediated cleavage which removes the glycan cap and mucin-like domains (Fig. 1C). These steps uncover critical residues for virus attachment to host cells, thus increasing the infectivity of the Ebola virions. We have previously shown the utility of flow virometry as a characterization tool for quantitative measurement of virus particles^20^. Here, we extend the application of flow virometry to development of an at-line PAT and evaluate conditions during the lab scale manufacturing of the recombinant VSV vaccine particles that may result in compromised VSV virions with reduced potency that remain undetected until performance of a release plaque assay. We show that using flow virometry to monitor the ERVEBO production process can enable process consistency and detect suboptimal process conditions much earlier than infectivity-based potency results. Furthermore, we describe a method to determine the cause for damage to the viral particles. Finally, we establish a direct correlation between damaged virus particles and reduced infectivity using a high throughput and high content overnight, imaging-based infectivity assay and offer this as an in-process infectivity assay with short turnaround time.

To further establish the general application of this PAT to other viruses, we used a human cytomegalovirus (HCMV) LVV candidate from MSD Research Laboratories. This is a conditionally replication-defective HCMV virus with restored expression of the pentameric complex and a characteristic particle size distribution^20^. Similar to ERVEBO, we find that flow virometry can detect changes in HCMV quality impacted by reagents or temperature variabilities in a simulated manufacturing process.

## Results

### rVSV-ZEBOV current manufacturing process description

**Figure 2:**
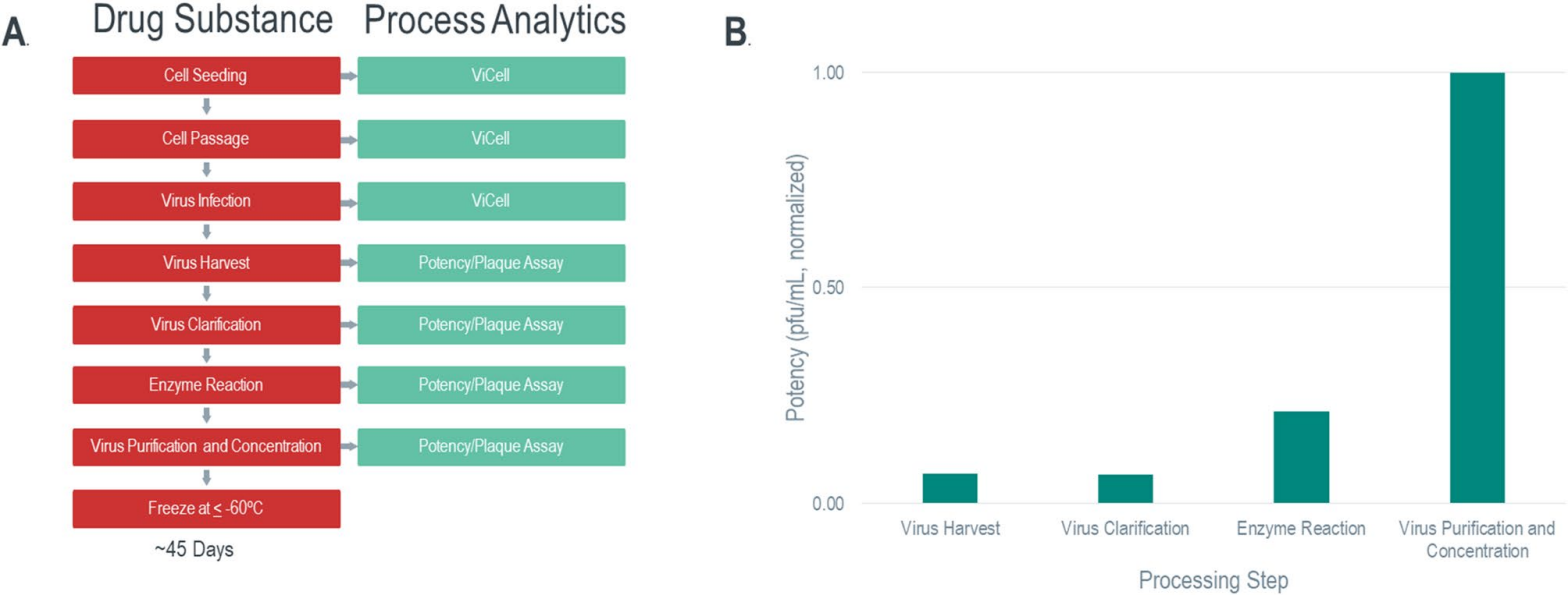
Ebola vaccine manufacturing upstream and downstream process description. (**A**) Upstream and downstream drug substance process steps (red) and corresponding analytics in use (green) for process monitoring/characterization. (**B**). Typical plaque based normalized potency assay results after each downstream process step for harvested virus fluid (virus harvest), clarified harvest (virus clarification), enzyme reaction and purified and concentrated virus. Potency shows a characteristic increase after the enzyme reaction and virus purification and concentration steps.

The current manufacture of ERVEBO consists of a cell growth phase, followed by viral infection and replication phase, harvest of viral fluids (HVF material), and clarification via depth filtration (Fig. 2A) as described before^28^. The clarified material is transferred to a vessel for treatment with recombinant Trypsin followed by purification and concentration to produce the bulk DS. The protease treatment creates a distinct conformational change in the surface proteins by clipping the Ebola GP1, removing parts of the surface proteins and increasing viral infectivity similar to the effects of natural cathepsin-mediated cleavage (discussed in^28^). Per manufacturer specifications, the activity of Trypsin is dependent on maintaining storage conditions, most notably to protect from light and extreme temperature (> 37 °C).

Current in-process analytics used for manufacturing ERVEBO include initial cell counts and MOI calculations for virus seeding and a plaque assay potency test on HVF harvest and samples taken before and after the enzyme reaction step (Fig. 2A). A change in potency value throughout the process is noted, whereby the HVF and clarified harvest (CH) (post depth-filtration) samples have about twofold lower potency than the enzyme reacted virus harvest (reacted virus harvest, RVH) (Fig. 2B). A further increase in potency is obtained after final purification and concentration of the virus (Fig. 2B).

### rVSV-ZEBOV characterization studies indicate temperature during enzyme reaction alters virus profile

The current ERVEBO manufacturing process involves transfer of the CH material to be heated at 37 °C in presence of recombinant Trypsin to generate RVH. To understand the impact of variability in key process parameters or raw materials on virus quality, we designed the following experimental arms : (i) extended hold time at room temperature of CH before enzyme reaction, (ii) increased heat (> 37 °C) during enzyme reaction, (iii) use of light exposed Trypsin (to potentially degrade activity) during enzyme reaction and (iv) performing heated reaction step without adding Trypsin to the reaction mix. For each arm of study, pre and post enzyme reaction material was measured by Simple Western (a high throughput and automated Western Blot method described in materials and methods) to detect GP1 cleaving and Apogee and Cytoflex flow virometers were used to determine particle concentrations and assess size distributions. All experimental conditions were compared to baseline (also referred to as “centerpoint”) representative control material.

**Figure 3:**
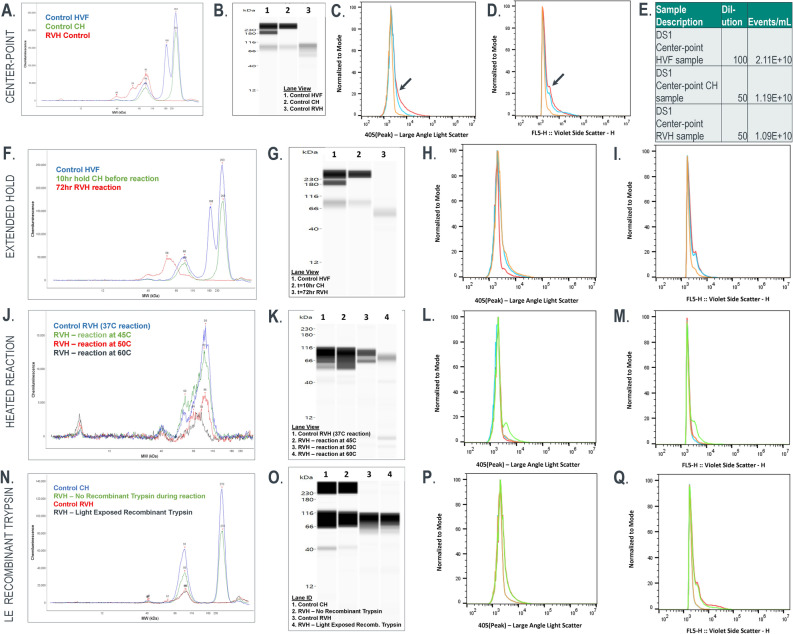
Detection of center-point and altered profiles for HVF, CH and RVH samples by Simple Western (SW) and flow virometry. (**A**) Representative Simple Western plots shown for center-point samples pre and post trypsin treatment. Pre-trypsinization, harvested viral fluids (HVF, blue line) and clarified harvest (CH, green line) probed with an anti-EBOV-GP polyclonal rabbit antibody show the presence of a > 230 kDa protein indicating the presence of intact GP1. Post trypsinization at 37 degrees C for 80 min, reacted viral harvest (RVH, red line) shows absence of the > 230 kDa protein and appearance of a lower molecular weight protein at ~ 100 kDa. (**B**) Corresponding lane view (computer-generated representative gel image) is shown for each SW plot in (A). (**C**) HVF (red line), CH (blue line) and RVH (yellow line) profiles for centerpoint samples run on Apogee. (**D**) HVF (red line), CH (blue line) and RVH (yellow line) profiles for centerpoint samples run on Cytoflex. Arrow indicates right shoulder in HVF and CH samples. (**E**) Total particle counts for HVF, CH and RVH centerpoint samples measured by Apogee. (**F**) Simple western detection of normal HVF (blue line), CH samples after extended hold at room temperature (green line) and corresponding RVH sample (red line). (**G**) Lane view of samples shown in (**F**). (**H**) HVF (yellow line), extended hold CH (blue line) and RVH (red line) samples run on Apogee. (**I**) HVF (red line), extended hold CH (blue line) and RVH (yellow line) samples run on Cytoflex. (**J**) Higher temperatures during trypsinization reaction performed at 45 (green line), 50 (red line) and 60 degree C (black line), as well as a control reaction run at 37 degree C (blue line), show absence of higher molecular weight protein seen in HVF and CH and presence of lower molecular weight molecule at ~ 100 kDa. (**K**) Lane view of samples shown in (**J**). (**L**) RVH samples generated at 45 degree C (blue line), 50 degree C (yellow line) and 60 degree C (green line) compared to centerpoint RVH (red line) ran on Apogee. (**M**) RVH samples generated at 45 degree C (yellow line), 50 degree C (red line) and 60 degree C (green line) compared to centerpoint RVH (red line) ran on Cytoflex. (**N**) Light exposed Trypsin (black line) showed similar GP1 cleaving activity as normal Trypsin (red line) on RVH samples, with both reactions showing the absence of the 230 kDa protein and presence of the cleaved lower molecular weight GP1. Not adding Trypsin during enzyme reaction step shows no GP1 clipping (green line) in RVH samples generated, similar to control CH sample (blue line). (**O**) Corresponding lane view for samples shown in (N). (**P**) Light exposed Trypsin treated RVH (yellow line) overlays with center point RVH (blue line) and no Trypsin added RVH (green line) overlays with centerpoint CH (red line) in Apogee. (**Q**) Light exposed Trypsin treated RVH (yellow line) overlays with center point RVH (blue line) and no Trypsin added RVH (green line) overlays with centerpoint CH (red line) in Cytoflex.

For control samples, we detected a > 230 kDa protein band in HVF and CH samples which was replaced by a ~ 100 kDa band in RVH samples (Fig. 3A, Simple Western profile and B, gel view). The virus profiles for HVF (red line) and CH (blue line) as characterized by Apogee (Fig. 3C) and Cytoflex (Fig. 3D) had a distinct right shoulder (arrow) in addition to the central peak, which disappeared in RVH samples (yellow line, Fig. 3C,D) that only had a single central peak. Apogee and Cytoflex both detected total virus particle counts of approximately 1e10/ml for HVF, CH and RVH samples respectively (Fig. 3E). For samples that had an extended hold time of CH (10 h) at room temperature prior to enzyme reaction, we found Simple Western to give the normal band patterns of > 230 kDa band in CH and replacement with 100 kDa band for RVH (Fig. 3F–G). Apogee and Cytoflex assessment of total particle count and profile did not reveal any significant variations from control samples in 3 C and D for this arm (Fig. 3H–I). This suggests that changes in either Mw or particle size distribution profiles are the results of clarification and enzyme reactivation step and not due to extended incubation. For samples that were heated to 45, 50 or 60 °C during the enzyme reaction step, we found that the RVH samples still showed the presence of the 100 kDa band and absence of the > 230 kDa band (Fig. 3J,K). However, increased smearing as well as reduced intensity of bands was noticed in the higher temperature samples, potentially due to generalized heat mediated degradation of the viral glycoprotein prior to running on gel (Fig. 3K). Both Apogee and Cytoflex results show a small but distinct feature on the right side of the profile in the RVH samples heated to 45 °C (Fig. 3L, blue line, Fig. 3M, red line). This became more pronounced and distinct with increasing temperature (Apogee, Fig. 3L, orange and green lines and Cytoflex, Fig. 3M, orange and green lines).

Use of light exposed Trypsin during the enzyme reaction step at the optimal temperature of 37 °C did not reveal any changes in the CH or RVH profiles by Simple Western (Fig. 3N–O) or by Apogee (Fig. 3P, orange line) and Cytoflex (Fig. 3Q, orange line). Finally, where CH was heated at 37 °C for 70 h to simulate the enzyme reaction step without adding Trypsin to the mix, Simple Western showed the continued presence of the > 230 kDa band in pre and post reacted samples (Fig. 3N,O), confirming the role of Trypsin in causing GP1 cleavage. Likewise, Apogee and Cytoflex showed profiles for post heated samples that looked identical to the control CH profiles with the right feature and not the control RVH profile (Fig. 3P,Q, red line).

### Temperature alterations compromise virus membrane integrity

**Figure 4:**
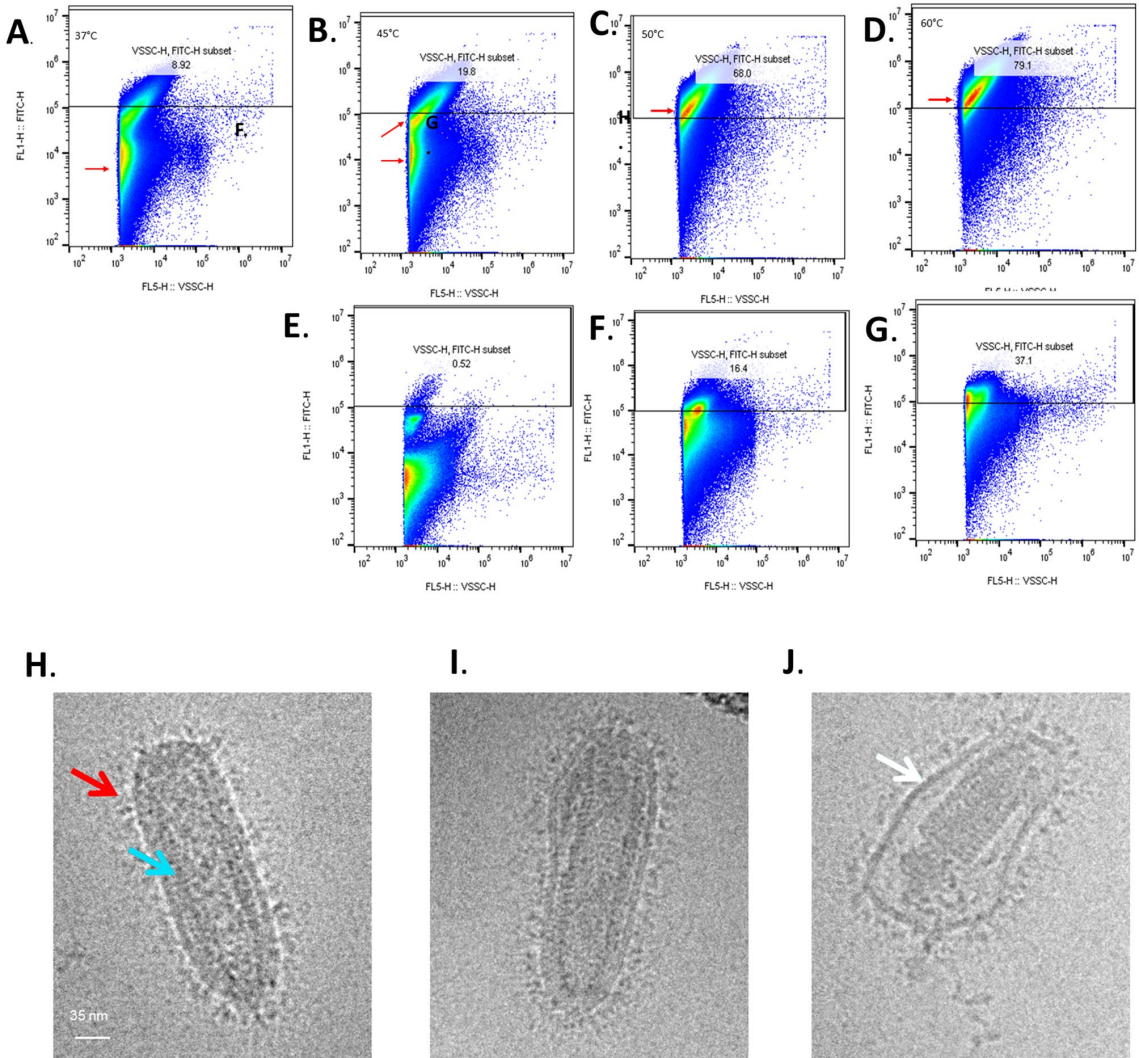
Detection of membrane damaged virus particles by intra-virus staining. Gating strategy for virus particles: violet laser side scatter (VSSC) defines virus population on X-axis and membrane staining with anti-VSV M and N antibody uses anti-mouse secondary AF488 to detect a positive signal on y axis. A gate for AF488 positive population was set based on unstained virus particles. Number indicated within this gate shows percent positive of total population of virions detected. (**A**) Center-point virus particles appear to have ~ 9% particles positively stained for anti-VSV antibody. As virus is heated, the anti-VSV positive population increases from 20% at 45° C (**B**), to 68% at 50° C (**C**) to 79% at 60° C (**D**). To confirm that pattern of VSV staining seen after heating is consistent with that induced by an established method for membrane permeabilization, center-point virions negative for anti-VSV (**E**) were treated with 0.05% and 0.5% Tween 20 (**F**,**G**) for 30 min at room temperature. In both instances, a positive population of virions appeared within the y axis gate, with percent positive increasing with increased concentrations of Tween. Electron microscopy imaging of center point CH samples shows typical bullet shaped particles (**H**) with distinct appearance of outer membrane with GP protein (red arrow) and nucleocapsid of virus (blue arrow). Heating the samples to 50° C for 10 min results in morphological changes in the appearance of virions (**I**) as well as clear separation of outer membrane (**J,** white arrow) from inner nucleocapsid.

To further understand the cause for the altered virus profile for heated RVH samples (at temperatures 45, 50, and 60 °C), we hypothesized that the appearance of the right shoulder could be due to distortion of individual virus particles and/or virus aggregation upon damage from heat. Since the right shoulder indicated particles of larger size causing increased light scatter, a key hypothesis was heat caused loss of viral membrane integrity resulting in virus particles sticking to each other to form aggregates. To test this hypothesis, we stained control and heated virus particles with an anti-VSV polyclonal antibody with no additional permeabilization of the virus particles. We reasoned that if the virus envelope was intact, the anti-VSV antibody, specific to intra-virus M and N proteins, will not be able to access these proteins and we would get a predominantly unstained, negative population in the flow profile. However, if the virus envelope was damaged the antibody would gain access to the internal virus proteins, resulting in an increase in the positively stained population. Using the previously described arm (ii) experimental samples, we stained and ran RVH samples generated at 45, 50, and 60 °C enzyme reaction on the Cytoflex. We found that control RVH samples had about 8% of anti-VSV antibody positively stained virus particles (Fig. 4A). RVH samples generated at 45 °C had about 20% virus particles that were stained for anti-VSV (Fig. 4B). Samples generated at 50 and 60 °C showed 68 and 79% positive populations, respectively (Fig. 4C,D). These studies confirmed the hypothesis that progressive heating of virus particles resulted in damage to the virus envelope and could explain the formation of the right shoulder. All heat treated samples used in this study were also tested using the gold standard plaque potency assay and showed a progressive reduction in potency upon heating (Supplementary Fig. 1).

To further demonstrate that anti-VSV antibody staining of rVSV-ZEBOV virions is directly related to virus envelope damage, we took control RVH samples and treated them with Tween 20, a known membrane damaging agent. We found that control RVH samples had a negligible population (0.52%) of virus particles that stained positively for anti-VSV (Fig. 4E). The treated population successively increased upon exposure to 0.05 and 0.5% Tween 20 to 16 and 37% total particles positively stained respectively (Fig. 4F,G). Finally, to visually evaluate the impact of heat on virus particles, we performed cryo-EM on pre- and post-heated CH ERVEBO samples. We found control CH samples visually represent the typical bullet shaped morphology with distinct and clearly visible layering of outermost GP proteins on virus membrane (Fig. 4H, red arrow) and inside nuclear capsid core (Fig. 4H, blue arrow). Upon heat treatment, an obvious change in virus morphology is seen with almost all virions being distorted in shape (Fig. 4I). In addition, heating frequently detaches the viral membrane from the inner virus capsid (Fig. 4J, white arrow) and a clear outer GP layer is no longer visible.

### Virus with compromised membrane shows reduced infection of Vero cells

**Figure 5:**
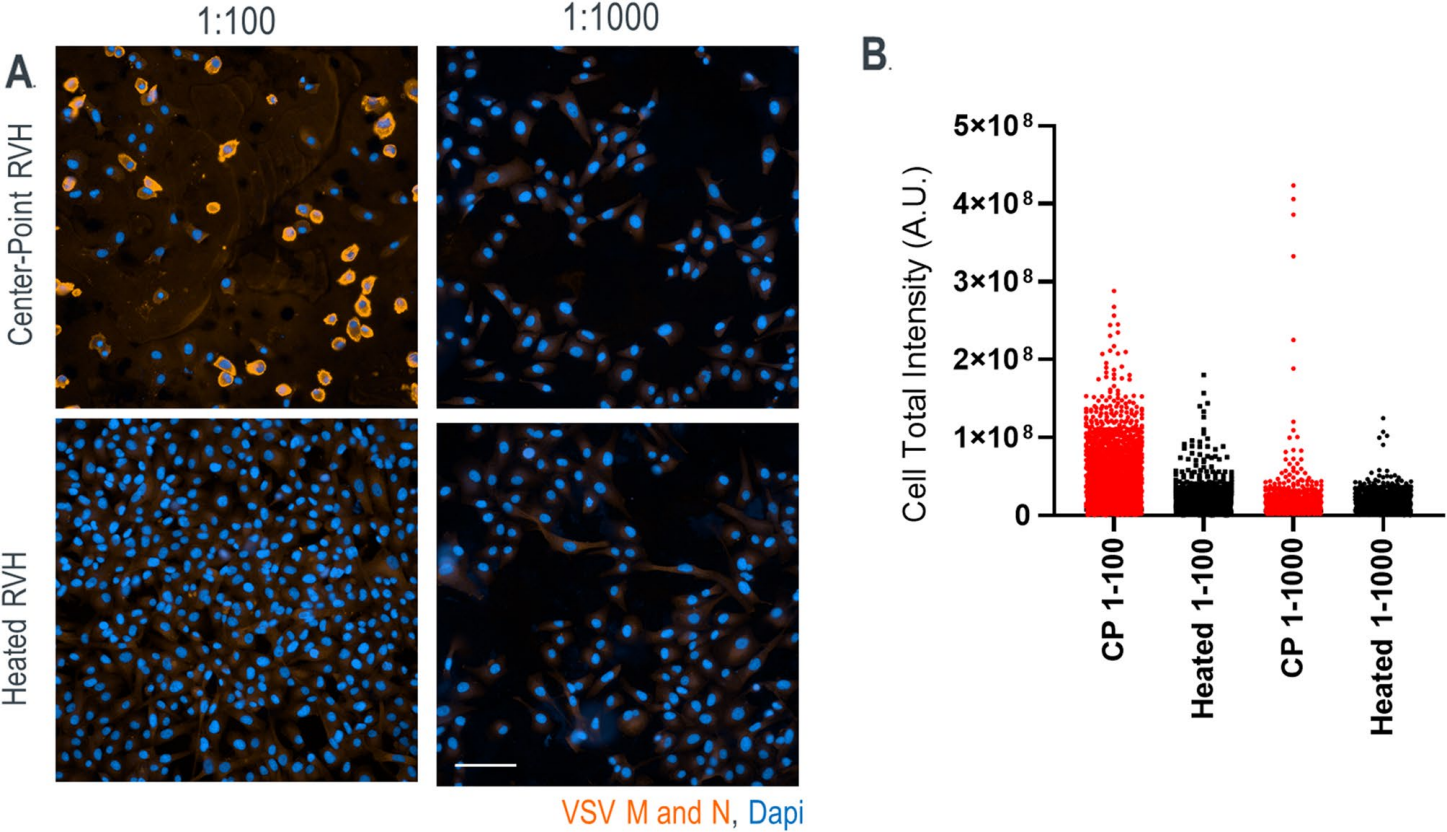
Overnight infectivity assay shows significantly reduced infection of Vero cells by heat damaged virus. (**A**) Representative images shown for Vero cells infected with center point RVH samples at a 1:100 and 1:1000 dilution (top panel left and right respectively) and cells infected with RVH generated at 50° C at 1:100 and 1:1000 dilution (bottom panel left and right respectively). Cells are stained with anti-VSV M and N polyclonal antibody (orange) and counterstained with Dapi (blue). (**B**) Quantitation of mean total sum intensity per cell for centerpoint and heated samples at 1:100 dilution and 1:1000 dilution. (*Due to increased cytopathic effect in cells infected with CP samples at 1:10 dilution, images for this set are not shown. **At least 10,000 cells per well were counted for each sample type and all dilution effects were tested in 2 column replicates).

The utility of a PAT to detect early virus particle damage in LVV manufacturing is limited unless also being able to perform a subsequent infectivity assay to evaluate any effect of the damage on potency. To establish a surrogate infectivity assay that can be performed at or near the manufacturing floor, we performed a 22 h infection of Vero cells with the control as well as heat-treated RVH samples. Dilutions of 1:10, 1:100 and 1:1000 for both control and treated RVH samples was done in duplicate columns of a 96 well plate seeded with Vero cells. We found evidence of significant cytopathic effect (CPE) and cell death in the wells infected with control RVH at 1:10 dilution. At the 1:100 dilution and 1:1000 dilutions of the control virus, greater than 80% of Vero cells were infected and stained positively for anti-VSV antibody (Fig. 5A, top panel). For the RVH samples generated at 50 °C, total cells infected and positive for anti-VSV antibody was between 20 and 50% (Fig. 5A, bottom panel). Quantitation of total fluorescence intensity detected from each infected cell for both control and heat treated RVH samples was performed to provide an indication of the level of virus replication in the cells. At least 10,000 individual infected cells were counted per sample type using high content imaging. An average total intensity value per cell of 1.4 x 10^7^ for control samples at the 1:100 dilution and 8.2 x 10^6^ at 1:1000 dilution was observed, with corresponding average intensity value of 6 x 10^6^ for heat treated samples at similar dilutions. A range of total intensity from 3x10^8^−1 x 10^4^ was seen for control samples at the 1:100 dilution. The corresponding range for heat treated samples diluted at 1:100 was 3 x 10^6^−200. For centerpoint samples at 1:1000 dilution the range of total intensity was 6 x 10^8^−2500 and the corresponding range for heat treated samples at the same dilution was 2 x 10^6^−250 (Fig. 5B). Statistical analysis using an unpaired two-tailed t test on log transformed data showed a P < 0.05 (highly significant) difference between the centerpoint and heat treated samples in total cell intensity for 1:100 dilution and P < 0.07 (moderately significant) difference for 1:1000 dilution.

### Flow virometry detects altered profile in HCMV virions subjected to process stress

**Figure 6:**
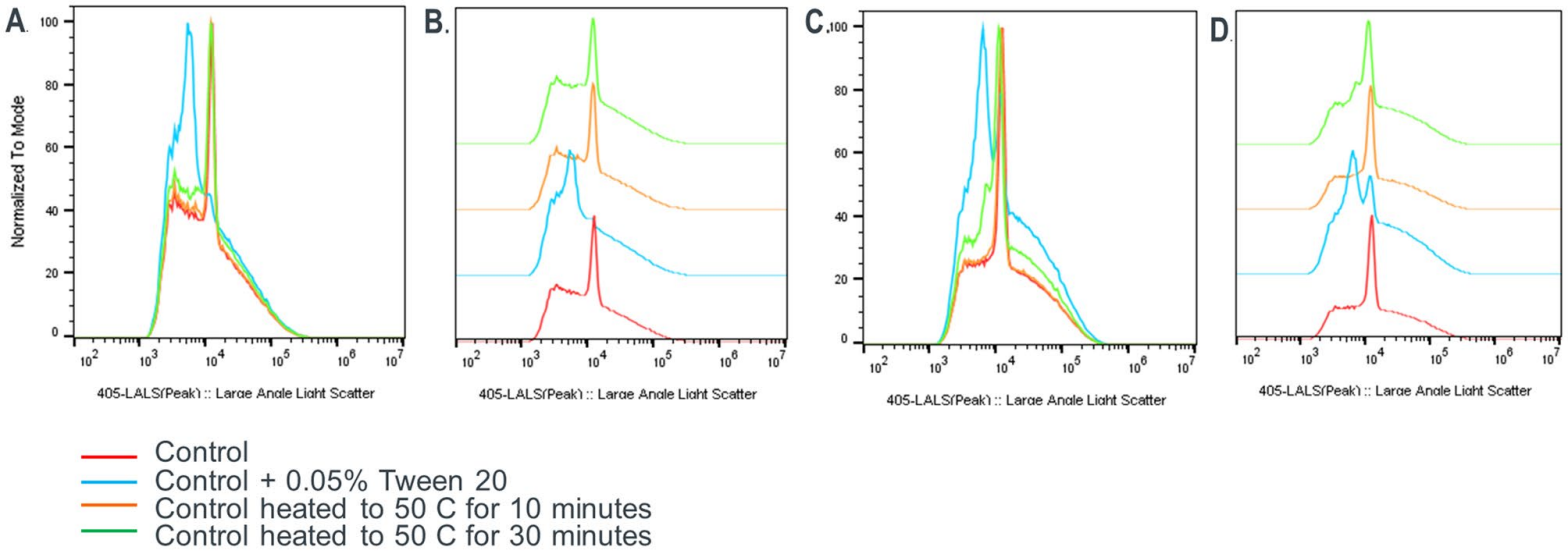
HCMV shows altered profile pattern upon membrane damage. (**A**) HCMV harvest derived “normal” profile (red line) measured by large angle light scatter in Apogee flow virometer shifts to the left upon treatment with 0.05% Tween 20 for 30 min at room temperature (blue line). A slightly less, but defined shift to the left is noticed upon heating DS at 50 C for 10 (orange line) and 30 (green line) minutes respectively. (**B**) Half offset staggered images of A shown to better visualize shift and alteration in profile shape. (**C**) HCMV partially purified process intermediate derived “normal” profile (red line) measured by large angle light scatter in Apogee flow virometer shifts to the left upon treatment with 0.05% Tween 20 for 30 min at room temperature (blue line). A slightly less, but defined shift to the left is noticed upon heating DS at 50 C for 10 (orange line) and 30 (green line) minutes respectively. (**D**) Half offset staggered images of C shown to better visualize shift and alteration in profile shape.

Flow virometry has previously been utilized to visualize the different populations of heterogeneous viral particles present in HCMV bulk substance^20^. A control HCMV HVF sample has a center peak along with a right and a left shoulder. The main peak includes the intact virions as well as non-infectious enveloped particles (NIEPs) in the sample, whereas the shoulders contain dense bodies (particles without capsids) of various sizes formed during HCMV replication. We applied heat and Tween 20 at out of range specifications to detect effects on the virus population. We hypothesized that significant changes in profile shape would occur which can be easily detected with an in-process monitoring tool, similar to the changes seen in ERBEVO. For this, control HVF HCMV samples derived from upstream processing were either treated with 0.05% Tween 20 for 30 min at room temperature or heated to 50 °C for 10 and 30 min respectively. Tween treatment showed a significant shift of the main center peak to the left (Fig. 6A,B, blue plot) whereas heat treatment resulted in the formation of a slightly larger left shoulder compared to control samples (Fig. 6A,B, orange and green plots). To test if these parameters also affected downstream samples generated post clarification, we performed similar treatment of a purified and concentrated HCMV sample (Fig. 6C,D). This downstream sample represent a different matrix than the harvested sample. The change in pattern seen in downstream samples was different and distinct from that seen in upstream samples. Tween treatment resulted in a shift to the left as well as multiple main peaks (Fig. 6C,D, blue plots). Heating for 10 min at 50 °C did not show significant changes (Figure C–D, orange plots), whereas heating for 30 min resulted in a shift of the main peak to the left as well as appearance of additional smaller peaks (Fig. 6C,D, green plots).

## Discussion

The power of PAT to directly monitor virus particles at crucial stages of large-scale manufacturing is its ability to provide enhanced process control and increase process robustness. In addition to providing improved control strategy, such tools, if applied correctly, can be used to avert expensive batch failures. However, flow virometry as a PAT to monitor LVV manufacturing in real time has thus far been an under-utilized application in industry. This is presumably because these tools are fairly new and significant characterization work still remains to be done to understand or establish their full potential. Unlike other more well-established PAT like Raman probes widely used for bio-process monitoring^29–31^, applications of flow cytometry have been limited to clinical use. Arguably, the long standing history of routine utilization of flow cytometers in rigorous clinical settings^32^ to identify and monitor disease and respond to treatment provides evidence of robustness of these platforms. And with the rapid expansion of the repertoire of LVVs, the need to evaluate and continuously expand the existing tool box that ensures innovative and advanced control strategies in large scale manufacturing process, is significant. From a cost perspective, commercial benchtop flow virometers are priced at or under a 100,000 USD and are reliable and easy to maintain. This pricing falls well within the range of other routine in-process analytical instruments used frequently in biopharma like high performance liquid chromatograms (HPLCs) and Raman probes. In addition, these instruments can function as standalone plug and play machines and do not require additional infrastructure, that can significantly increase cost. Some of the logistical pros and cons of using these newer process monitoring tools compared to gold standard potency assays is outlined in Supplementary Table 1.

Our study represents a successful application of high throughput flow virometry as a potential PAT to detect conformational changes and subtle virus particle degradation in two unrelated LVV products: the recently approved ERVEBO and the HCMV LVV candidate. In ERVEBO, we focus on the effects of temperature on virus particle quality. Heating is a critical step required in the manufacturing of this product as it results in clipping of the Ebola GP1 protein by protease (here recombinant Trypsin). However, heating also introduces a point of vulnerability in large scale manufacturing and requires precise temperature control over extended time. Here we provide a control strategy around evaluating the effect of variability in heat directly on virus particles. We show that heating at or around 45 °C or higher during the enzyme reaction step can start modifying morphology of a fraction of virions and appears as a small but measurable right shoulder in the virus profile. It is possible that the conformational change in protein upon GP1 clipping also alters refractivity or optical density of the particles so that the light path through the virions is collectively altered and detected by flow cytometry as an altered profile shape. If applied as a PAT on a manufacturing floor, the flow virometry assay used here to detect initial morphological changes in-process can then possibly trigger a corrective measures and save the batch or future batches. The assay is quick and easy and can be done at-line with only microliters of sample, no additional processing and within ten minutes. Samples were run fresh and frozen and no significant differences in counts or profiles were observed between these two, underscoring the ease of use and flexibility of this method. For ERVEBO, a slightly advanced version of the same method was used to further probe the cause for unusual RVH profile in heated samples by staining the virions with virus specific antibodies. The hypothesis is that an intact virion will be impermeable to staining with antibodies against intra-virus proteins without any additional permeabilization steps. Our results showed selective staining of damaged particles with increased heating. Cryo-EM imaging at high resolution showed profound morphological differences between normal and heated virus particles. Though not a high-throughput method, such imaging tools are invaluable in the direct visualization of otherwise “invisible” particles. Traditional plaque potency assays confirm the loss in potency seen using both flow virometry and quantitative imaging assay. It is perhaps not surprising to see an aggravated loss in plaque potency for particles heated at 50 °C, though the percent population of damaged virus is less than a hundred percent at this temperature. This could be a combination of plaque assay sensitivity not being able to detect differences that are within assay noise as well as flow virometry being unable to discriminate between individual “damaged” infectious particles contributing to potency loss versus aggregates of particles that may represent a higher percent damaged population than is currently shown.

In addition to developing a potential PAT for virus quality measurement, we have also performed a surrogate, imaging-based infectivity assay that can be used for in-process assessment of virus potency. The high content and high throughput quantitative imaging platform used here offers the distinct advantage of being able to automatically count and quantify thousands of cells within minutes as well as can be multiplexed with additional cellular markers for virus infection. Combined with the PAT for direct virus monitoring, these two technologies can provide virus potency measurements much before release assays and be utilized for further control over continuous bioprocess monitoring.

Using HCMV as a model enveloped virus particle, here we explore the effect of two stressors, heat and Tween 20, on virus quality. Tween 20 is a detergent commonly added to buffers and reagents for ELISA chemistry to act as a surfactant and emulsifier. If the concentration of Tween is not carefully monitored, it can destroy lipid bilayer membranes such as those on enveloped viral particles. In our study, this property is used to simulate sample contamination with chemical residues or constituents which could potentially degrade the enveloped particles and result in low potency. We find that heating and Tween 20 treatments, both done for thirty minutes, have separate and distinct effects on the characteristics of enveloped virus particles, demonstrated by alterations in virus profile. The effects seen on upstream and downstream samples are also distinct, potentially due to differences in matrices, where the upstream samples have more cellular material and debris. Both Tween and heat cause the HCMV profile to shift to the left, but the shift is much more pronounced with Tween. With heat, an increase in height of the already existing “left shoulder” in HCMV upstream samples is seen over time, but a complete shift of the center peak of virions and NIEPs is not observed. Presumably, these differences indicate both the extent and kinetics of virus particle damage.

As treatment with Tween 20 is harsh, the membrane damage is more severe. Heating causes the same damage, but the kinetics is slower. The difference in profile for same treatments on downstream samples is intriguing. Here, both Tween and heating for 30 min seem to have a more severe effect on virions with the appearance of multiple peaks previously not seen, potentially suggesting sample degradation. As downstream samples are much more concentrated than upstream material and also exist in a very different matrix that is devoid of protection from cell culture media and serum, this result is not unexpected. These studies demonstrate the sensitivity of flow virometry that can differentiate not only between samples based on the specific process stress, but also between the various stages of manufacturing process (upstream or downstream) and can be simply and effectively integrated into existing workflows. No additional sample processing, small sample volumes and quick sample running time remain key enablers to this method.

LVVs are manufactured worldwide and manufacturing programs exist over many decades. Evaluation of the longevity and product life cycle management of the tool, is therefore, an important aspect in the selection of a new PAT. Currently, multiple flow virometry platforms are being tested for application to small particle measurement including exosomes and virus particles^33–36^. To mitigate concerns around a specific platform, we were able to show proof of concept studies using two independent flow virometers that both show the same effect on virus particles, and can both be suitable PAT.

Finally, and in conclusion, it is critical that fit for purpose analytics are designed to provide near real time monitoring of LVVs in large-scale manufacturing. The goal in doing this is twofold. One, it is significantly cost saving to be able to characterize product quality as early in the process as possible in order to intervene with corrective measures. Second, it is important to be able to have robust process monitoring tools to evaluate the impact of large-scale changes in raw materials on virus seed stocks and production. For many LVV processes it is challenging to assess the effects of a change in poorly characterized raw materials such as serum on product quality and studies implemented to characterize such process and raw material changes are lengthy and may be difficult to interpret. The integrated tools represented in this study provide an advancement opportunity for the rapid and robust monitoring and control of large molecule therapeutics.

## Methods

### Virus source

All virus samples used in this study were derived in MSD laboratories and used in lab scale studies.

### Scale-down studies to generate rVSV-ZEBOV samples

All virus samples used in this study were generated using scaled down models of the commercial-scale process. Samples were stored at < − 60 °C after generation and thawed at room temperature for testing or further manipulation. Sample treatment conditions with Tween 20 and heat are listed in sample names and were performed immediately prior to testing. The Tween was added in the PBS dilution buffer to specified concentrations and sample thermal treatments were performed in a temperature-controlled heating block.

### Simple western assay

The Simple Western assay was run on an automated capillary western blotting instrument produced by ProteinSimple. The instrument emulates the typical western blot workflow in a capillary tube, including separation by molecular weight, treatment with primary and secondary antibodies, and detection by chemiluminescence. This method has been described in^28^.

Prior to analysis by capillary western blot, ERVEBO samples were diluted up to 5 × depending on anticipated concentration and then added to a mixture containing sodium dodecyl sulfate (SDS), dithiothreitol (DTT), and fluorescent molecular weight standards. After a 10-min incubation at 70 °C, samples are added to a 12–230 kDa sample plate along with 1000 × anti-ZEBOV GP-rabbit primary antibody, anti-rabbit-HRP secondary antibody, and luminol-peroxide.

#### Instrument settings

Separation time was set to 30 min, primary antibody incubation was set to 45 min, and secondary incubation time was set to 45 min. Detection performed on chemiluminescent channel. All analysis performed at 2 s exposure time.

### Apogee and cytoflex assays

The Apogee A60 is configured with 405 nm violet laser and forward, side, and green florescence detectors. Apogee refers to traditional forward and side scatter as small-angle light scatter (SALS) and large-angle light scatter (LALS) respectively. The Cytoflex S is in standard configuration with three color lasers and assorted detectors; for the purposes of this study we are concerned primarily with violet side scatter (VSSC, analogous to the Apogee 405 nm LALS). No customization was required of the equipment after initial set up.

Highly filtered sheath fluid; either 0.1 µm deionized water, 0.12 µm Milli-Q water, or purchased Beckman Coulter sheath fluid, were used for all of these studies. Both cytometers were confirmed to be capable of visualizing and enumerating control beads and ERVEBO viral particles with control samples prior to using for experimental conditions.

HVF, CH and RVH were diluted in 0.1 µm filtered PBS and run on Apogee and Cytoflex Flow Virometers, dilutions are noted in the sample names on Figures. For Apogee, a flow rate of 1.5 ul/minute was selected and for Cytoflex a flow rate of 10ul/minute was used. Total particle count for samples was measured by multiplying events/second recorded with dilution of the sample and total volume collected during each run. The profile of each sample was recorded and exported as fcs files to plot using FlowJo software.

Control polystyrene beads for confirming operation of flow cytometers were sourced through Apogee and utilized per manufacturer recommendations. The Apogee A60 utilizes a traditional photomultiplier tube (PMT) with variable voltage, settings were identical for all profiles which we compared to one another. The laser wattage was set to 270mW to increase resolution for ERVEBO viral particles and set to 70mW for the larger, heterogenous population of HCMV. The Cytoflex S does not utilize a PMT based system in favor of avalanche photodiodes, and laser wattage is not user-variable; our unit was set to 92mW on initial install.

### Membrane damage staining

CH and RVH samples from control and heated runs were diluted 100X in 0.1 micron filtered PBS and stained with anti-VSV rabbit polyclonal antibody for 30 min at room temperature. The samples were then clarified by passing through CaptoCore column once and then stained with anti-rabbit AF488 secondary antibody for 30 min at room temperature. After this, the samples were once more clarified by CaptoCore column purification and run directly on Cytoflex. To detect membrane damage, positive signal from AF488 was detected on y axis from virus particles gated on violet laser side scatter (VSSC) on x axis.

### 24-h infectivity assay

Control and experimental RVH samples were used to infect Vero cells plated overnight in 96 well plates. A serial dilution of viruses in Vero cell media was performed to obtain a dose response infectivity curve. 24 h post infection, virus containing media was removed and cells were permeabilized and fixed using BD Perm Fix solution directly in 96 well plate. Staining of virus in cells was then performed using anti-VSV rabbit polyclonal antibody (Imanis) at a 1:1000 dilution for one hour at 37degC. Secondary staining was performed with anti-rabbit AF568 secondary at 1:200 dilution for 40 min at 37degC. A DAPI counterstain was applied and plates were imaged using Perkin Elmer Operetta CLS high content imager. Image analysis was performed using the Harmony software (PE). Statistical analysis was done on log transformed data using Graph Pad Prism software. A P value of < 0.05 was considered highly significant. P values between 0.1 and 0.05 were considered moderately significant.

## Supplementary Information

Supplementary Information
