# Supplementary material for: Flow virometry for process monitoring of live virus vaccines-lessons learned from ERVEBO
Source: Sci Rep. 2021 Apr 1;11:7432. doi: 10.1038/s41598-021-86688-z (PMC8016999; doi:10.1038/s41598-021-86688-z)
Supplement: Supplementary file 1 — Supplementary Information [file 41598_2021_86688_MOESM1_ESM.docx]

**Flow virometry for process monitoring of live virus vaccines-lessons learned from ERVEBO**

Geoffri Ricci^1^, Kevin Minsker^1^, Austin Kapish^1^, James Osborn^1^, Sha Ha^1^, Joseph Davide^1^, Joseph P. Califano^1^, Darrell Sehlin^1^, Richard R. Rustandi^2^, Lawrence W. Dick Jr. ^2^Josef Vlasak^2^, Timothy D. Culp^3^, Andreas Baudy^4^, Edward Bell^1^, Malini Mukherjee^1^.

^1^ Vaccines Process Development and Commercialization, Merck & Co., Inc., West Point, PA, USA

^2^ Vaccines Analytical Research and Development, Merck & Co., Inc., West Point, PA, USA

^3^Vaccines Process Development, Merck & Co., Inc., West Point, PA, USA

^4^Safety Assessment and Laboratory Animal Resources, Merck & Co., Inc., West Point, PA, USA

*Corresponding author:

Malini Mukherjee, PhD

770 Sumneytown Pike,

WP 42-3

West Point, PA 19486, USA

Email: [malini.mukherjee@merck.com](mailto:malini.mukherjee@merck.com)

|  | **Flow Virometry Method** | **Traditional Plaque or TCID50 Cell-Based Assays** |
| --- | --- | --- |
| Preparation Work | Use commercially available PBS | Vial thaw cell bank, serially passage to testing levels, then continue to maintain cell line throughout testing. |
| Number of Operators | One to two operators required to dilute samples and run the equipment | Typically a minimum of 4 to 6 operators to maintain cell trains, passage for testing,  perform multiple replicates, and generate results like counting plaques/TCID cytopathic effect |
| Lead Time | Assuming samples are run fresh from process sampling, sampling and testing times are >5 minutes per replicate | Excluding preparation work, cell growth phase (2-5 days), infection phase (5-20 days), and results generation (hours for custom automated equipment, days for manual counting) |
| Equipment | Commercial flow cytometers/virometers available with low capital investment (< 300K USD) | For manual methods, typical lab equipment (microscope, CTUs, etc.), for automated results generation, custom instruments are required (> 500K USD for equipment, more for development) |
| Variability | Variability based on sensitivity of instrument and operator dilutions, typically < 10% for standard flow cytometry | Sources of variability include cell metabolic cycles, reagent effects on cells (serum, etc.), operator dilutions, subjective reading of plaques or CPE, combined assay variability typically > 20% |
| Responsiveness | Testing can be performed nearly at-line with in-process samples, allowing for responding to signals potentially during batch execution | With longer lead times, multiple production batches may be committed before an issue is detected |
| Throughput | Low processing time increases throughput, approximately can run ≥ 20 samples in an hour | High replicate requirements to overcome variability lower throughput in addition to long processing times |

**Supplementary Table 1.** Table summarizes key differences between in-process flow virometry assays and traditional potency assays used for live virus vaccines.


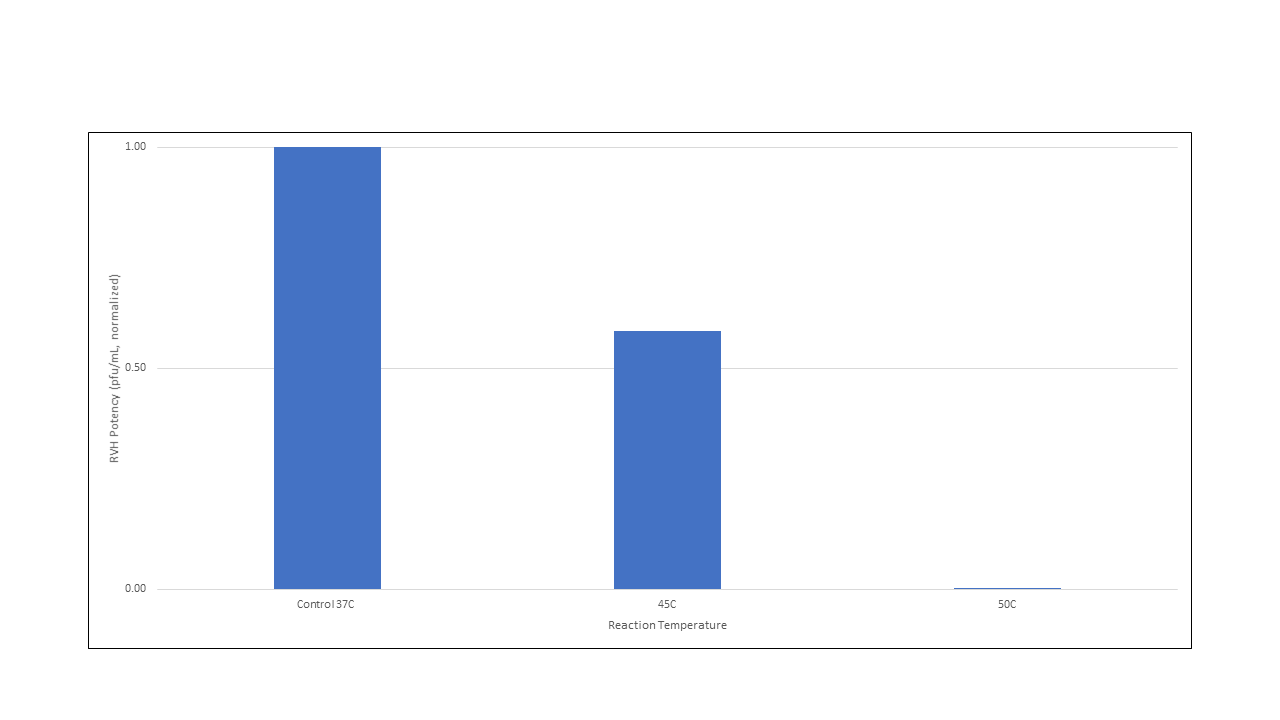


**Supplementary Figure 1. Heat treated rVSV-ZEBOV virus particles show decrease in plaque potency.**

**(A).** RVH potency data normalized to the control (37°C) enzyme reaction result. A significant reduction in potency is observed when the reaction is run at 45°C, and an even greater reduction is seen with a 50°C reaction. RVH produced from a 60°C enzyme reaction was also tested for plaque potency assay, but that condition did not return a result above the quantifiable limit.
